# Intestinal Tumorigenesis Is Not Affected by Progesterone Signaling in Rodent Models

**DOI:** 10.1371/journal.pone.0022620

**Published:** 2011-07-27

**Authors:** Jarom Heijmans, Vanesa Muncan, Rutger J. Jacobs, Eveline S. M. de Jonge-Muller, Laura Graven, Izak Biemond, Antwan G. Ederveen, Patrick G. Groothuis, Sietse Mosselman, James C. Hardwick, Daniel W. Hommes, Gijs R. van den Brink

**Affiliations:** 1 Department of Gastroenterology and Hepatology, Leiden University Medical Center, Leiden, The Netherlands; 2 Tytgat Institute for Liver and Intestinal Research, Academic Medical Center, Amsterdam, The Netherlands; 3 Merck, Sharpe and Dohme, Women's Health Department, Oss, The Netherlands; 4 Department of Gastroenterology and Hepatology, Academic Medical Center, Amsterdam, The Netherlands; National University of Singapore, Singapore

## Abstract

Clinical data suggest that progestins have chemopreventive properties in the development of colorectal cancer. We set out to examine a potential protective effect of progestins and progesterone signaling on colon cancer development. In normal and neoplastic intestinal tissue, we found that the progesterone receptor (PR) is not expressed. Expression was confined to sporadic mesenchymal cells. To analyze the influence of systemic progesterone receptor signaling, we crossed mice that lacked the progesterone receptor (*PRKO*) to the *Apc^Min/+^* mouse, a model for spontaneous intestinal polyposis. *PRKO-Apc^Min/+^*mice exhibited no change in polyp number, size or localization compared to *Apc^Min/+^*. To examine effects of progestins on the intestinal epithelium that are independent of the PR, we treated mice with MPA. We found no effects of either progesterone or MPA on gross intestinal morphology or epithelial proliferation. Also, in rats treated with MPA, injection with the carcinogen azoxymethane did not result in a difference in the number or size of aberrant crypt foci, a surrogate end-point for adenoma development. We conclude that expression of the progesterone receptor is limited to cells in the intestinal mesenchyme. We did not observe any effect of progesterone receptor signaling or of progestin treatment in rodent models of intestinal tumorigenesis.

## Introduction

The Women's Health Initiative (WHI) was launched in 1991 to conduct medical research into some of the major health problems of older women. Among other studies, the WHI performed two large prospective randomized clinical trials where postmenopausal hormone use was evaluated. One trial consisted of treatment with estrogens combined with the progestin medroxyprogesterone acetate (MPA) versus placebo, to evaluate the risk of endometrial carcinoma [1], [2]. In the second trial estrogen alone was compared to placebo in women that had previously undergone a hysterectomy [1], [3]. A substantial 40% risk reduction (*P* = 0.003) for colon cancer development was observed in women that received the combination therapy [4], whereas the risk of colorectal cancer was slightly but not significantly increased by treatment with estrogens alone.

Based on these results, progestins have been suggested as putative chemopreventive agents for colon cancer [5], [6], however, the mechanism of action by which they work in the intestine remains obscure.

Progesterone signaling plays multiple roles in the physiology of the female body. Perhaps best known for its important function as a mitogen for endometrial tissue [7] and regulator of the mammary stem cell development [8], it also has pleiotropic effects on many other physiological functions. For example progesterone signaling attenuates osteogenesis [9] and increases sexual receptivity [10]. Also, progesterone reduces the immune response of the uterine environment [11], and diminishes cytokine production by plasmacytoid dendritic cells [12], [13].

All major effects of progesterone are thought to be mediated by the progesterone receptor (PR), a member of the nuclear receptor superfamily. This receptor, has at least two isoforms (the PR-A and PR-B), that have distinct effects [14]. Although signaling by progesterone is mediated by the PR exclusively [15], Progestins (synthetic progesterone receptor ligands) can have off-target effects at high concentrations or dosages, that are mediated by other steroid hormone receptors such as the androgen receptor (AR), the estradiol receptors (ER) or the glucocorticoid receptor (GR), depending on the progestin used.

Some investigators have reported that normal colonic tissue as well as colorectal cancers express PR mRNA [16] or show progesterone binding capacity [17]. In contrast, work by others suggests that few colon cancers express PR and expression is low [18] or find no evidence for PR expression, at least in the epithelium [19]. PR expression has been documented in colon cancer cell lines and data on growth inhibition of these cell lines by progestins points towards a role for progestins as being antiproliferative [20]. No studies comment on the function of PR in mesenchymal cells, whereas the role of mesenchymal cells in colon cancer is of emerging importance [21]–[23].

In this study we sought to elucidate the role for the progesterone receptor and progestins in colorectal carcinoma development.

## Results

### The progesterone receptor is not expressed in the epithelium of the small intestine or colon

**Figure 1:**
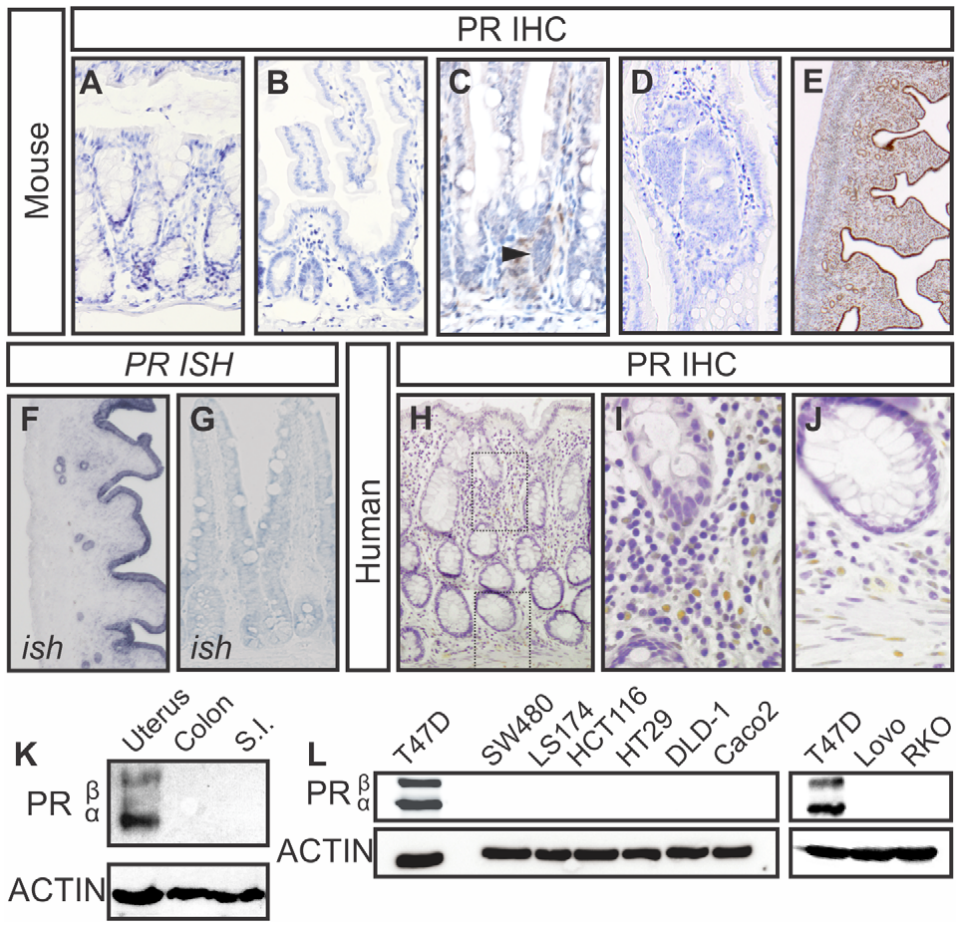
Progesterone Receptor expression in mesenchymal cells in the intestine, not in the epithelium. A–E) PR immunohistochemistry on the mouse colon (A) and small intestine (B,C) where rare cells express PR (arrowhead). And in an adenoma of an *Apc^Min/+^* mouse (D). PR is widely expressed in the mouse uterus (E). F,G) *In situ* hybridization in mouse uterus (F) and small intestine (G). All murine tissue shown was taken from A female animal in diestrous stage, when progesterone is high [39]. H–J) PR Expression in the human colon is located in mesenchymal cells (I) and the smooth muscle layer (J), similar to the mouse intestine. For *in situ* hybridization, thick (10 µm) sections were used, which makes identification of mesenchymal cells difficult. K) PR immunoblot on the mouse colon and small intestine. L) PR immunoblot on a panel of colon cancer cell lines shows no expression of either PR-A or PR-B isoform. The breast cancer cell line T47D is used as a positive control.

Hypothesizing that the progesterone receptor is the main mediator of the effect of progestins, we set out to examine the expression of the progesterone receptor in the epithelium of the small and large intestine (Fig. 1). Since expression of PR in some tissues is known to vary greatly during stages of the estrous cycle [14], we analyzed tissue of female mice in all stages of the estrous cycle as well as male mice (data of male mice not shown). To avoid issues of detection level and antibody specificity, we tested multiple antibodies (see Table 1 for antibody information, data not shown), and confirmed the results with mRNA *in situ* hybridization. We observed no detectable epithelial expression of the PR at either the mRNA or protein level (Fig. 1A–K). In contrast, some PR positivity was observed in rare cells in the lamina propria (Fig. 1C). All antibodies reacted with PR in the mouse uterus, which was used as a positive control (Fig. 1E) but not with the uterus of mice that lack the PR (PRKO mice, data not shown). To further confirm absence of PR in normal tissue, we performed immunoblots on lysates of mouse colon and small intestine, using the uterus as a positive control. Both isoforms of the PR were highly present in the uterus, but we found no expression of the PR in both colon or small intestine (Fig. 1K).

**Table 1:** Antibodies used for immunohistochemical detection of PR.

Company	Antigen	Clone	Raised in animal	Antigen retrieval	Dilution used
ABR	PR	MA1-410	Rabbit polyclonal	Citrate	1∶400
Dako	PR	A0098	Rabbit Polyclonal	Citrate	1∶400
NeoMarkers	PR	SP2	Rabbit Monoclonal	Citrate	1∶200
NeoMarkers	PR	AB13	Rabbit Polyclonal	Citrate	1∶1000
Roche	BrdU	BMC 9318	Mouse Monoclonal	Citrate	1∶200

We next analyzed PR expression in adenomas of *Apc^min/+^* mice. These mice carry a mutation in the *Apc* gene that resembles oncogenic mutations in patients with the Familial Adenomatous Polyposis syndrome and in most sporadically occurring colorectal carcinomas (Su et al., 1992). *Apc^Min/+^* mice develop multiple polyps in the small and large intestine and due to their resemblance with human colorectal adenomas, they are widely used as a model for human colorectal cancer [24]–[26]. In adenomas of *Apc^min/+^* mice we found that the PR was expressed by rare lamina propria cells but not by epithelial cells (Fig. 1D).

Immunohistochemical analysis of PR expression in the human colon was similar to expression we found in the mouse. In human mucosa, PR positive cells were observed in the mesenchyme such as leukocytes and smooth muscle cells but no epithelial expression was detected (Fig. 1 H–J). This was the case in normal colonic epithelium as well as adenomas and carcinomas (data not shown). As it was previously suggested that colon cancer cells may express PR [20], we next examined a number of different colon cancer cell lines for expression of PR at the protein and RNA level and using the T47D breast cancer cell line as a positive control. In a panel of 6 frequently used colon cancer cell lines, we found no evidence of PR expression at either protein (Fig. 1L.) or RNA level (not shown)).

### No effect of progesterone signaling or progestins on intestinal epithelial proliferation or tumorigenesis

**Figure 2:**
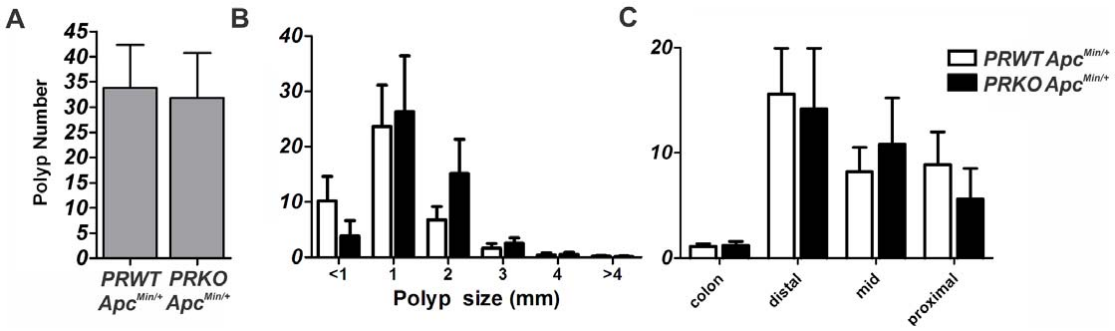
Lack of off-target effects from progestins on intestinal proliferation or development of acfs. A) Treatment of a panel of colon cancer cell lines with MPA or progesterone (P4) has no effect on viability at relevant concentrations. B) BrdU incorporation in small intestine or colon after challenging female animals with MPA or progesterone (P4) for four consecutive days. C–E) Acf count in the Azoxymethane treated rat shows acf number (C), localization of acfs throughout the colon (D) and multiplicity (E) (number of crypts per acf).

Since no PR was detectable in colon cancer cell lines, PR- mediated signaling is not possible in these cells. At high concentrations progestins, such as MPA that was used in the WHI study, bind to steroid hormone receptors other than the PR [27]. Such off-target effects might be important in the protective role of progestins. To investigate a possible off-target effect of MPA or progesterone directly on colonic epithelial cells, we treated all cell lines that were previously tested negative for expression of PR, with increasing concentrations of these steroids (Fig. 2A). To prevent interference from steroids that are present in high concentrations in FCS, we charcoal stripped our serum prior to use. Additionally we used medium that was phenol red free, since this has weak estrogenic capacities [28]. Measuring viability of all cell lines, no effects were seen treating with concentrations up to 200 ng ml^−1^, which is approximately 10 times higher than physiological plasma levels of progesterone or than levels of MPA that are achieved with contraceptive [29].

**Figure 3:**
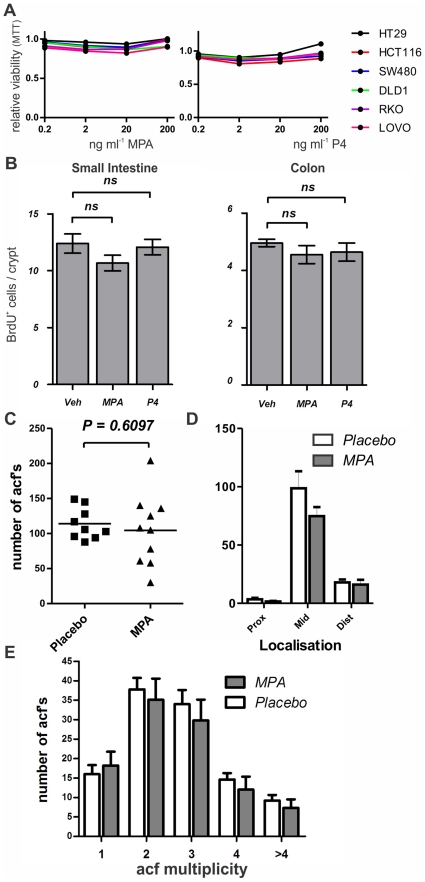
The Progesterone receptor has no influence on intestinal polyposis. A–C) Development of spontaneous polyposis in the *Apc^Min/+^* mouse is not altered by *PRKO* (10 female animals per genotype).

Even though intestinal epithelial cells may not express PR and do not seem to be affected by progestins, these cells may be indirectly affected by PR signaling in adjacent cells in mesenchyme. Also, it has been reported, that tumorigenesis can be influenced indirectly, via systemic or central effects [30]. We therefore decided to cross the *Apc^Min/+^* mouse to a *PRKO* background to examine whether systemic absence of the PR affects intestinal adenoma development in mice. Analysis of both tumor number and size did not yield differences between *PRKO* and WT mice. Also, localization of tumors throughout the intestine and colon was not different (Fig. 3 A–C).

Although we did not observe any effect of *PRKO* on the development of adenomas in the *Apc^Min/+^* mouse, this does not exclude potential off-target signaling, mediated by other receptors. Hypothesizing that progestins, such as MPA, harbor effects that are independent from the PR, we decided to examine the effect of daily physiological doses of MPA (4 mg kg^−1^) and progesterone (32 mg kg^−1^) on epithelial homeostasis in the normal mouse and the effect of MPA (1 mg kg^−1^) on aberrant crypt focus formation in azoxymethane treated rats.

First, we examined if proliferation of epithelial cells was influenced by administration of MPA or progesterone. We treated female mice for 4 days with these hormones, and counted the number of BrdU positive cells per crypt (Fig. 2B). No difference in proliferation was found between animals that were treated with MPA, progesterone or vehicle (corn oil), respectively. Also, there were no gross changes in intestinal architecture or in differentiation of distinct epithelial cell types, as could be judged on sections of intestine of these mice, stained with Haematoxylin and Eosin.

To investigate the possibility that progestins exert their effect on dysplastic cells rather than normal epithelium, we treated rats with slow release pellets that contained either MPA or vehicle, and induced colonic tumorigenesis by injecting these animals with the carcinogen azoxymethane. This agent causes DNA mutations in epithelial cells, that lead to development of aberrant crypt foci (ACFs) [31]. ACFs are hyperproliferative crypts, of which is thought that a proportion develops into polyps and later into carcinomas [32], [33].

There was no change in the numbers of ACFs in placebo-treated versus MPA-treated animals. Also, no effect was found on the localization of these ACFs throughout the colon, or on the multiplicity, (i.e. the number of crypts of which an ACF consists) (Fig. 2C–E).

## Discussion

Progestins reduced the risk of colorectal cancer in a large randomized prospective study in postmenopausal women [2], [4]. We have analyzed expression of the PR and function of the PR and of Progestins in colorectal cancer models. We find that although there are rare mesenchymal cells that express the PR in the lamina propria, the PR is not expressed in either normal or malignant intestinal epithelium or in colorectal cancer cell lines. We do not observe any effect of either progesterone or MPA on intestinal epithelial homeostasis or rodent models of intestinal tumorigenesis.

The expression of the PR was previously demonstrated in whole tissue RNA of both normal colon and colorectal cancer samples [16], [18]. Slattery and colleagues subsequently found no evidence for PR expression in the epithelium of either normal colon or colon cancer samples using immunohistochemistry [19]. More recently expression of PR was described in HT29 and HCT116 colon cancer cell lines and the same authors described inhibition of the proliferation of these cell lines by MPA.

Our findings corroborate those of Slattery and colleagues as we find no evidence for PR expression in the epithelium of normal small intestine or colon in humans and mice. Also, we did not detect any PR in either human colon cancer cell lines, human samples of colorectal cancer, in mouse adenomas or aberrant crypt foci in the rat. Our findings suggest that the PR mRNA that was found in whole tissue by others may have been derived from PR positive cells present in the lamina propria or from intestinal smooth muscle cells. Using the T47D breast cancer cell line as an appropriate positive control for PR expression we find no evidence of PR expression at either mRNA or protein level in any of the colon cancer cell lines examined. In accordance with the absence of PR expression from intestinal epithelium and colon cancer cell lines we did not find any effect of either progesterone or MPA on the proliferation of either normal intestinal epithelium *in vivo* or colon cancer cell lines *in vitro*.

We were subsequently unable to find a role for progesterone signaling in initiation or progression of intestinal adenomas in the *Apc^Min/+^* mouse or on aberrant crypt formation in azoxymethane-treated rats.

In the WHI studies it was found that the combination of MPA plus estrogen had chemopreventive effects on colon cancer development whereas estrogens alone had no effect. Since treatment with MPA as a single drug has no role in postmenopausal women, it was never examined. Our studies clearly show that progesterone signaling alone does not affect rodent models of intestinal tumorigenesis, nor were we able to find any off-target effects of Progesterone or MPA.

Although it may be possible that the combination of estrogen and MPA may affect colorectal cancer, this does not seem very likely in light of the absence of PR expression in either normal colon or colorectal cancer and lack of effect of *PRKO* on *Apc^Min/+^* adenomas. Rodent models for colorectal cancer do often not progress to the post-adenoma stage (e.g. adenocarcinoma and metastasis). Our studies thus can not rule out a role in late stages of colorectal tumors for either progestin monotherapy or a combination with estrogens.

In conclusion, our studies do not support a role for either progesterone or MPA signaling homeostasis of normal colonic epithelium or in colon cancer development.

## Methods

### Animal experiments

All experiments were performed according to the Leiden University Medical Center animal experimental committee guidelines. Animal experiments were approved by the animal experimental committee (DEC) of the animal research facility (PDC) of the Leiden University Medical Center under approval numbers 08138 and 08145.

Wild type rats and mice were obtained from The Jackson Laboratory (Bar Harbor, MN, USA) or from our own breeding facility. *Apc^Min^* animals [34] were obtained via The Jackson Laboratory. *PRKO* mice [35] were bred heterozygously into *Apc^Min/+^* males. Male animals that were heterozygous for both alleles were bred into females, heterozygous for the PRKO allele to generate females that were *PRKO Apc^Min/+^*.

For the Rat azoxymethane experiment five week old rats were ovariectomized and slow release pellets with MPA or vehicle (Innovative Research of America, Sarasota, FL, USA) were implanted subcutaneously in the neck. These pellets contained 25 mg of MPA in a 90 day slow release pellet. Vehicle pellets were of the same size and composition, but contained no steroids. After surgery and implantation of the pellet, rats were left to acclimatize for one week prior to injection with azoxymethane.

Subsequently, rats were injected twice with azoxymethane (10 mg kg^−1^ day^−1^; Sigma-Aldrich, Zwijndrecht, Netherlands) with 7 days between the two injections. Six weeks after the first injection, animals were sacrificed and colons were fixed.

To assess proliferation *in vivo*, BrdU incorporation studies were performed. Six weeks old mice were injected on four consecutive days MPA (Sigma-Aldrich), Progesterone (Sigma-Aldrich) or vehicle (10 animals per group). Hormones were dissolved in DMSO which was subsequently diluted to 10% in corn oil. All volumes were equivalent. One hour prior to sacrifice, all animals were injected with 200 µl BrdU (10 mg ml^−1^ in PBS; Sigma-Aldrich).

### Hormone treatment

For rat studies, the MPA dosage used was based on the IC50 of ovulation-inhibition in rats (0,1 mg kg^−1^ day^−1^) [36], to guarantee physiologically active concentrations, this concentration was multiplied tenfold (1 mg kg^−1^ day^−1^). Rats received a 90 day slow release pellet to ensure stable release. This was approximated by placement of a pellet containing 25 mg of MPA (1,1 mg kg^−1^ day^−1^).

For mouse BrdU incorporation studies, the concentration of MPA was multiplied by 4 to ensure allosteric conversion between rats and mice [37]. The concentration of progesterone was based on the report of Yamanouchi et al. [38], where estrogen induced proliferation in rats was inhibited by treatment with 8 mg kg^−1^ day^−1^ progesterone. Allosteric conversion resulted in treatment with 32 mg kg^−1^ day^−1^ progesterone in mice.

### Tissue processing, Immunohistochemistry and *In situ* hybridization

Tissue was fixed in 10% ice-cold formalin embedded in paraffin. Sections of 4 µm were deparaffinized in xylene and rehydrated.

For immunohistochemistry, endogenous peroxidase was blocked using 0.3% H_2_O_2_ in Methanol. The sections were cooked in 0.01 M Citrate buffer pH 6.0 for 20 minutes and incubated with the primary antibody in PBS with 1% BSA and 0.1% Triton X-100.

Antibody binding was visualized with Powervision HRP labeled secondary antibodies, and diaminobenzidine for substrate development. All sections were counterstained with Mayer's haematoxylin. For a list of all antibodies used, see Table 1. Immunohistochemistry in figures was done with the rabbit monoclonal antibody from Neomarkers (clone SP2), since the background was low using this antibody.

For *in situ* hybridizations, sections were deparaffinized and rehydrated. Subsequently, sections were incubated in 1 M HCl for 10 minutes, treated with proteinase K in PBS for 20 minutes, and refixed with 4% paraformaldehyde for 10 minutes. Sections were acetylated with acetic anhydride, and incubated with a digoxigenin (DIG)-labeled probe over three nights at 68°C. After three stringency washes with 50% formamide in SSC buffer (pH 4.5) at 65°C, sections were incubated with alkaline phosphatase-labeled anti-DIG Fab fragments (Roche). Probe binding was visualized using the NBT-BciP substrate (Sigma-Aldrich).

### BrdU and aberrant crypt focus (acf) counting

For BrdU incorporation studies, sections were stained as described above. Blinded to treatment group, the number of BrdU^+^ cells was counted in at least 30 crypts in each animal (n = 10 per group).

For counting of aberrant crypt foci (as), fixed colons were stained with 1% methylene blue (Sigma-Aldrich) in PBS and washed in fresh PBS. Acf number and multiplicity was evaluated in the entire colon under a dissection microscope.

### Immunoblotting

Cells and tissue were lysed in cell lysis buffer (Cell Signaling Technology, Leiden, Netherlands). Protein concentration in lysates was assessed by bicinchoninic acid protein assay reagent (Pierce, Thermo scientific, Etten-Leur, Netherlands). Lysates were boiled in sample buffer containing 0.25 M Tris-HCl pH 6.8, 8% SDS, 30% glycerol, 0.02% bromophenol blue and 1% β-ME. Separation was done on 10% SDS-PAGE, and proteins were transferred to a PVDF membrane. Specific detection was done by incubating the blot overnight in TBS with 0.1% Tween-20 with 1% BSA with anti-PR (NeoMarkers 1∶500) and anti-Actin (1∶2000; Santa Cruz Biotechnology, CA, USA) antibodies. Antibody binding was visualized using the Lumi-Light western blotting substrate (Roche).

### Charcoal stripping of fetal calf serum

Five grams of charcoal (Merck) was put into 50 ml of FCS and left overnight on a rollerbank at 4°C. The charcoal was pelletted by spinning at 5000 rpm subsequently and the serum was decantated and filtered through a 0,22 µ filter.

### Cell Culture and MTT

Cells culture was maintained in DMEM, supplemented with 10% FCS and 1% penicillin and streptomycin. For the MTT assay, cells were plated onto 96 well plates in phenol red free DMEM F12, supplemented with 5% Charcoal stripped FCS and 1% penicillin and streptomycin. Cells were left to adhere overnight. Per condition, 10 wells with cells were treated with the indicated concentration of either MPA or Progesterone (Sigma-Aldrich), dissolved in 100% ethanol. In all conditions, a final concentration of 1% ethanol was maintained. After 48 hours of treatment, thiazolyl blue tetrazolium blue (MTT) substrate (Sigma-Aldrich) was added to each well (5 mg ml^−1^ end concentration), and incubated for 4 hours. Culture medium containing excess MTT was taken off, and cells were lysed in isopropanol. MTT was measured colorimetrically at 570 nm. The average of all 10 wells per condition was taken as the outcome of one experiment.

Concentrations used in in vitro studies were based on reported medical reference values: in healthy cycling females, progesterone concentrations range from 1–20 ng ml^−1^. Progesterone plasma concentration is maximally 90 nanomole l^−1^ (approximately 30 ng ml^−1^) in healthy females. MPA plasma concentrations, 5–20 days after injection of a standard contraceptive dose (consisting of an intramuscular injection of 150 mg MPA) ranges from 10 to 25 ng ml^−1^ [29].

### Statistical analysis

All data are presented as mean ± standard error of the mean. Cell culture experiments were repeated at least three independent times. Statistical analysis of cell culture experiments was performed by 2-way ANOVAs analysis.

For animal experiments, student T-Test or 1-way ANOVAs tests were used. In sub analysis of localization or size, 2-way Anova tests were used. All Anova tests were followed by Bonferroni's post test for multiple comparisons.
