# Correction: Intestinal Tumorigenesis Is Not Affected by Progesterone Signaling in Rodent Models

**DOI:** 10.1371/annotation/b60d4ec5-4c6f-43ab-9f63-322e3cd59636

**Published:** 2013-06-05

**Authors:** Jarom Heijmans, Vanesa Muncan, Rutger J. Jacobs, Eveline S. M. de Jonge-Muller, Laura Graven, Izak Biemond, Antwan G. Ederveen, Patrick G. Groothuis, Sietse Mosselman, James C. Hardwick, Daniel W. Hommes, Gijs R. van den Brink

There was an error in the Funding statement. The correct version is:

This study was funded with grant UL-2010-4667 from the Dutch Cancer Society and with an unrestricted grant from Merck, Sharpe & Dohme (MSD). P.G.G., A.G.E. and S.M. are researchers employed by MSD. They have contributed to this study by critical appraisal of the data and the manuscript and by sharing expert opinion. Furthermore, MSD had no role in study design, data collection and analysis, decision to publish or preparation of the manuscript. 

